# Novel Mode of nanoLuciferase Packaging in SARS-CoV-2 Virions and VLPs Provides Versatile Reporters for Virus Production

**DOI:** 10.3390/v15061335

**Published:** 2023-06-07

**Authors:** Rebekah C. Gullberg, Judith Frydman

**Affiliations:** Department of Biology, Stanford University, Stanford, CA 94305, USA; rgullber@stanford.edu

**Keywords:** COVID-19, SARS-CoV-2, VLP, virus egress, coronavirus, virus reporters, nanoLuciferase assay, antivirals, drugs screens, virus assembly

## Abstract

SARS-CoV-2 is a positive-strand RNA virus in the Coronaviridae family that is responsible for morbidity and mortality worldwide. To better understand the molecular pathways leading to SARS-CoV-2 virus assembly, we examined a virus-like particle (VLP) system co-expressing all structural proteins together with an mRNA reporter encoding nanoLuciferase (herein nLuc). Surprisingly, the 19 kDa nLuc protein itself was encapsidated into VLPs, providing a better reporter than nLuc mRNA itself. Strikingly, infecting nLuc-expressing cells with the SARS-CoV-2, NL63 or OC43 coronaviruses yielded virions containing packaged nLuc that served to report viral production. In contrast, infection with the flaviviruses, dengue or Zika, did not lead to nLuc packaging and secretion. A panel of reporter protein variants revealed that the packaging is size-limited and requires cytoplasmic expression, indicating that the large virion of coronaviruses can encaspidate a small cytoplasmic reporter protein. Our findings open the way for powerful new approaches to measure coronavirus particle production, egress and viral entry mechanisms.

## 1. Introduction

Severe Acute Respiratory Syndrome Coronavirus 2 (SARS-CoV-2) belongs to the family *Coronaviridae*, which are the largest positive-strand RNA viruses that infect humans. SARS-CoV-2 is highly transmissible with a broad tissue tropism that is rapidly evolving in human populations. The enormous effort to develop countermeasures against SARS-CoV-2 has produced vaccines and antivirals that can reduce viral morbidity but have limited ability to prevent infection and curtail viral transmission. Research into viral mechanisms necessitates work under cumbersome biosafety level 3 (BSL3) conditions [1,2]. The large size of the coronavirus genome also makes viral reporters difficult to clone and engineer, hindering assays for drug screens.

The importance of developing better sensors and systems to study the coronavirus cannot be understated. In the last 20 years, three separate global coronaviruses outbreaks have spilled over into human populations, causing extensive morbidity and mortality: SARS-CoV [3,4], MERS-CoV [5], and now SARS-CoV-2 [6]. These highly transmissible and pathogenic viruses are expected to continue emerging and re-emerging in human populations, necessitating expanded rapid tools to improve efforts in diagnostic and therapeutic developments.

Coronaviruses are enveloped viruses with a ~30 kb positive-sense single-stranded RNA genome. Viral particles are ~100 nm in diameter and canonically composed of the spike (S), envelope (E), membrane (M), and nucleocapsid (N) [7,8] viral proteins. Despite extensive research, we still have a limited understanding of the mechanisms and pathways of viral particle assembly and egress at a cellular level of the coronavirus [8,9]. Model systems of viral assembly and egress can be useful tools to fill in gaps in our understanding. Virus-like particles (VLPs) provide commonly used tools to study viral assembly and egress, permitting studies in BSL2 conditions, even for pathogenic viruses. VLPs are composed of viral structural proteins overexpressed in cells that are necessary and sufficient to produce particles that physically resemble the virus of interest, but do not carry the genome, and thus are incapable of initiating an infection. VLP systems have applications to understand assembly and egress, as well as entry and neutralization, and can also provide effective vaccine platforms [10,11]. Many VLP platforms for SARS-CoV-2 have been developed by co-expressing the structural proteins in various cell types and ratios as tools to study assembly and entry [12,13,14,15,16,17,18,19]. A VLP system that harnessed a putative viral RNA packaging signal (PS9) was recently reported [12]. However, this reporter is highly inefficient, yielding 3–5% GFP positive cells after pseudo-infection with concentrated VLPs [12], and showed little difference compared to controls (Supplemental Figure S1A). In addition, SARS-CoV-2 recombinant systems carrying genetically encoded reporters have been developed, but the large size of the genome makes such recombinant efforts highly cumbersome [20,21]. Furthermore, the production of genetically encoded reporters for each new SARS-CoV-2 strain, as well as for emerging coronaviruses, is highly limited by the need to clone and engineer their very large viral genomes to produce infectious cDNAs. These considerations highlight the need for new concepts and improved coronavirus reporter platforms.

Here, we set out to build out a reporter virus-like particle (VLP) system to improve and expand the repertoire of available tools for more accessible BSL2 research of SARS-CoV-2. We exploited the phage-derived MS2 coat protein and MS2-binding loop interaction system to efficiently incorporate mRNAs encoding GFP and nanoLuciferase reporters into VLPs. Our characterization of this system led us to the surprising finding that the small (19 kDa) nanoLuciferase (herein nLuc) [22] protein itself was packaged into SARS-CoV-2 VLPs and secreted from cells. Interestingly, the nLuc protein was also packaged into the virus particles produced by the SARS-CoV-2, NL63 and OC43 coronaviruses, but was not secreted upon production of the dengue flaviviruses or the Zika virus, nor was it packaged into dengue VLPs. Our data shows that the large virion of coronaviruses can encaspidate and secrete a small cytoplasmic reporter protein. Strikingly, interventions or drugs that impair SARS-CoV-2 replication coordinately reduce both viral production and nLuc secretion, demonstrating the potential of our findings to produce versatile coronavirus infection reporters. Our findings open the way to using these two powerful approaches to measure coronaviral particle production and egress and viral entry mechanisms, both in BSL2 (for VLPs) and BSL3 (for WT virus) regimes.

## 2. Materials and Methods

### 2.1. Plasmid Production

The SARS-CoV-2 VLPs are composed of four main plasmids encoding the structural proteins that come from the codon optimized plasmid library of the SARS-CoV-2 viral proteins that was a gift from Nevan Krogan [23]. To make the original SARS-CoV-2 VLPs, we used pTwist-EF1alpha-nCoV-2019-S-2xStrep, pLVX-EF1alpha-nCoV2019-E-IRES-Puro, pLVX-EF1alpha-nCoV2019-M-IRES-Puro, and pLVX-EF1alpha-nCoV2019-N-IRES-Puro. To generate a more stable spike protein on the VLPs for the binder pull-down experiment, we inserted two standard mutations into the spike protein: the furin cleavage site aa682-685RRAR->GSAS, and the 2P site aa986-987KV->PP. Each set of mutations was added sequentially via a site directed mutagenesis kit with PfuUltraII (Agilent) following the manufacturer’s protocol and fully sequenced. The Dengue virus 2 VLP plasmid was a kind gift from Dr. Ted Pierson [24].

To modify the plasmids, we used a Gibson Assembly (ThermoFischer Scientific, Waltham, MA, USA) and fully sequenced them prior to use. Briefly, a PCR was performed to amplify each fragment (vector and insert) of the desired plasmid using PfuUltraII (Agilent) according to the manufacturer’s protocol. The PCR reactions were treated with DpnI (New England Biolabs, Ipswich, MA, USA) at 37 °C for 1 h (hr) and the expected size band of DNA was extracted from a 1% agarose gel with a GeneJet kit (ThermoFischer Scientific, Waltham, MA, USA). The two fragments were added to a Gibson Assembly reaction mix (ThermoFischer Scientific, Waltham, MA, USA) and incubated at 50 °C for 1 h followed by transformation into NEB Stable Competent *E. coli* cells, which were selected on LB agar plates with ampicillin or kanamycin. Three colonies of each plasmid were grown overnight in 5 mL cultures, and Minipreps (Qiagen, Hilden, Germany) were performed to isolate the DNA, which were fully sequenced with Primordium full plasmid sequencing, and the correct plasmid was grown in a 50 mL culture for a Qiagen MidiPrep prior to use in cells.

To generate the N-MS2 construct, a gene block encoding the sequence of MS2 (UniProt) was ordered from IDT, amplified by PCR, and added to the C-terminus of N after a 2XGSS linker via a Gibson Assembly. To generate the reporter mRNA with 12 copies of the MS2 binding loops (MBL), we started with our pEGFP-N1 backbone and sub-cloned in 12 copies of the V6-MBL [25]. We then exchanged the GFP for a nanoLuciferase via a second Gibson Assembly reaction and thus had both pEGFPN1-12xMBL and pnLuc-12MBL as our reporter mRNAs. To generate the tags on nLuc, we started by cloning nanoLuciferase into the pCDNA3.1 vector already containing a 3XGSS N-terminal linker and an ALFA tag via a Gibson Assembly. We then swapped the ALFA tag for a second nLuc sequence or a 1× NES or 3× SV40 NLS tag. The villin “headpiece” protein (VHP) construct was a kind gift from Dr. Ron Kopito [26], and either one copy or two copies were added C-terminally and separated by another GSS linker. Finally, we added an N-terminal Maltose binding protein (MBP) followed by a green fluorescent protein (GFP) to the nLuc plasmid via a Gibson Assembly generating the MG-nLuc plasmid. pLVX-EF1alpha-GFP-2xStrep-IRES-Puro was used as a control. All plasmids were fully sequenced to validate their identity and proper orientation after cloning.

### 2.2. Expression and Purification of SARS-CoV-2 VLPs

#### 2.2.1. Cells

VLPs were produced in adherent HEK-293T [293T] (ATCC-CRL-3216) or the suspension Expi293 cells (Gibco, ThermoFischer Scientific, Waltham, MA, USA). HEK-293T cells were maintained in 10% fetal bovine serum (Atlanta Biologicals. Flowery Branch, GA, USA) in Dulbecco’s Modified Eagle’s Medium (DMEM; Gibco, ThermoFischer Scientific, Waltham, MA, USA) with 1% penicillin–streptomycin (Gibco, ThermoFischer Scientific, Waltham, MA, USA) and 1% non-essential amino acids (neAA; Gibco, ThermoFischer Scientific, Waltham, MA, USA) at 37 °C in a humidified incubator with 5% CO_2_. Expi293 cells were maintained in Expi293 Expression Medium (Gibco, ThermoFischer Scientific, Waltham, MA, USA) at 37 °C in a humidified incubator with 8% CO_2_ shaking at 130 revolutions per minute.

#### 2.2.2. Transfection

HEK-293T cells were transfected with the indicated plasmids mixed at a 1:2 (μg DNA: μL transfection reagent) ratio with Lipofectamine2000 (Invitrogen, Carlsbad, CA, USA) in OpitMEM (Gibco, ThermoFischer Scientific, Waltham, MA, USA) following the manufacturer’s protocol. The Expi293 cells were transfected with the indicated plasmids at a 1:2 (μg DNA: μL transfection reagent) ratio with the ExpiFectamine 293 reagent, following the manufacturer’s instructions.

#### 2.2.3. VLP Collection

The VLP producing cell supernatants and cell pellets were collected after 48 h and processed. The supernatants were clarified at 1000× *g* for 10 min at room temperature, then filtered through a 0.45 μM PVDF membrane (Millipore Sigma, Burlington, MA, USA) to remove cell debris.

#### 2.2.4. VLP Concentration and Purification with Sucrose Cushion Ultracentrifugation

To concentrate and purify proteins in a higher density complex such as a VLP, the samples were pelleted through a 20% sucrose in a 120 mM HEPES cushion at 100,000× *g* for 2 h at 4 °C with 45 Ti or a 70 Ti fixed-angle rotor (Beckman-Coulter, Brea, CA, USA), depending on the volume of the sample. After centrifugation, the supernatants were removed, the ultracentrifuge tubes were inverted for 5 min on a paper towel to remove the remaining supernatant, and the pellets were gently resuspended in a TNE buffer overnight at 4 °C, and an equal volume was processed for loading onto a density gradient, western blotting, or nanoLuciferase activity experiments.

#### 2.2.5. Gradient Purification of VLPs

To biochemically characterize the density of the VLPs and further purify them, we ran Potassium Tartrate density gradients. Briefly, after concentration and an initial purification through a 20% sucrose cushion, the resuspended pellet of VLPs was loaded on top of a hand-poured step-gradient consisting of (from top to bottom): 5–40% K-tartrate with 3.75–30% glycerol in a TNE buffer. The gradients were centrifuged at 200,000× *g* for 3 h at 4 °C in a SW-41Ti rotor. The gradients were carefully removed and fractions were collected from the top by slowly pipetting 1 mL fractions. Each fraction was resuspended in 10 mLs of TNE and re-concentrated by ultra-centrifugation at 200,000× *g* for 3 h at 4 °C in a SW-41Ti rotor.

### 2.3. Western Blotting

To quantify the structural proteins in the gradients, 10 μL of VLPs, OC43 virions, or a mock supernatant were denatured with 2× Laemmli buffer (2% SDS, 10% glycerol, 0.002% bromophenol blue, and 0.06125MTris-HCl) with 5% β-mercaptoethanol at 50 °C for 10 min and loaded onto a 4–20% Tris-glycine SDS-PAGE gel (Invitrogen, Carlsbad, CA, USA). Similarly, cells expressing the nLuc constructs were lysed with 20 mM HEPES [pH 7.5], 100 mM NaCl, 1 mM EDTA, 1% Triton X-100 + protease inhibitors for 30 min on ice, clarified by centrifugation at 4 °C for 10 min at 16 kxg. The protein concentration was measured via BCA (ThermoFischer Scientific, Waltham, MA, USA), and 10 μg of protein was denatured with 2× Laemmli buffer containing 5% β-mercaptoethanol at 50 °C for 10 min and loaded onto a 4–20% Tris-glycine SDS-PAGE gel (Invitrogen, Carlsbad, CA, USA). The proteins for each gel were transferred to a nitrocellulose membrane with an iBlot (ThermoFischer Scientific, Waltham, MA, USA), which was blocked with 3% BSA in TBS containing 0.2% tween-20 (TBS-T) and probed with primary antibodies: StrepTagII (Abcam, rabbit, polyclonal), nanoLuc (Promega, mouse, monoclonal), HCoV-OC43 nucleocapsid (SinoBiological, rabbit, polyclonal), CD81 (Santa Cruz, mouse, monoclonal), or GAPDH (GeneTex, rabbit, polyclonal) overnight at 4 °C with gentle rocking. The membranes were then washed 3 times in TBS-T and incubated with IRE-dye-800—conjugated anti-rabbit or IRE-dye-680—conjugated anti-mouse antibodies at 1:10,000 dilution for 1 h, washed again three times in TBS-T, followed by three washes in DI water and imaged on the LiCor scanner.

### 2.4. RNA Extraction and qRT-PCR

RNA was extracted from the cell supernatants using Trizol LS (ThermoFisher Scientific, Waltham, MA, USA) following the manufacturer’s protocol. Primers were designed using Benchling primer design software for nsp15, EGFP, full-length nLuc-12xMBL, and the NCBI-nucleotide primer design tool for GAPDH (Table 1). A one-step qRT-PCR kit with SYBR green (Agilent) was used with the primer sequences listed below. Reactions were set up according to the manufacturer’s protocol and run on a BioRad CFX96 Touch Real-Time PCR Detection System. The cycling parameters were as follows: 20 min at 50 °C for reverse transcription, then 5 min at 95 °C, followed by 45 two-step cycles of 95 °C for 5 s and 60 °C for 60 s, followed by a melt curve starting at 65 °C and ending at 97 °C. The annealing/extension step was extended to 5 min at 60 °C for the full-length RT-PCR, which was separated on a 1% agarose gel and imaged under UV.

The qRT-PCR of VLPs in the supernatant was performed using VLPs co-expressed with the EGFP-12xMBL reporter RNA. The RNA was extracted following the indicated processing and the qRT-PCR was run with the EGFP primers below. Delta Cq values were calculated by subtracting the measured Cq value from a blank control or a cut off of 30 cycles.

To quantify the copies of SARS-CoV-2 genomic RNA in the supernatant, a standard curve composed of 10-fold dilutions of in vitro transcribed RNA from a SARS-CoV-2 cDNA plasmid (nucleotides: 19785-20364) was generated. The copies of RNA were calculated by measuring the concentration of RNA on a NanoDrop (ThermoFischer Scientific, Waltham, MA, USA) and converting it to the number of RNA copies with the NEB calculator by first converting them to moles of ssRNA: moles ssRNA (mol) = mass of ssRNA (g)/((length of ssRNA (nt) × 321.47 g/mol) + 18.02 g/mol), and then to RNA copy number with RNA copy number = moles of ssRNA × 6.022 × 10^23^ molecules/mo. The Cq value was measured in 10-fold dilutions of RNA and converted to a log value to generate the standard curve equation: Y = −2.467 × X + 31.25 (primer efficiency 154%), which was used to calculate the log copies of RNA in the qRT-PCR reaction, which was back-calculated to obtain copies/mL in the supernatant.

### 2.5. Microscopy

HEK293T cells were transfected with the indicated plasmids following the above protocol. After 24 h, the cells were transferred to poly-L-lysine (Sigma-Aldrich) coated glass chamber slides. After another 24 h, the cells were fixed in 4% paraformaldehyde for 15 min at room temperature. The cells were then permeabilized in 0.5% TritonX and blocked with 3% BSA. The primary antibody was nanoLuc (Promega, mouse, monoclonal), diluted 1:150 in PBS with 1% BSA with 0.1% TritonX and incubated overnight at 4 °C. Following incubation, the chambers were washed 3 times in PBS with 1% BSA and 0.01% TritonX, and then incubated with the secondary antibodies conjugated to AlexaFluor647 (1:500, ThermoFisher Scientific, Waltham, MA, USA) for 1 h at RT followed by three more washes including Hoechst (1:10,000 in PBS, ThermoFischer Scientific, Waltham, MA, USA) and overlaid with 50% glycerol. The cells were imaged with a Spectral Diskovery Spinning Disk microscope at 63× magnification, where z-stacks of the entire cells were collected and merged with a max projection using the FIJI (version for Mas OS X) software [27].

### 2.6. nanoLuciferase VLP Measurement

NanoLuciferase (nLuc) was measured following the manufacturer’s instructions. Briefly, the cells were plated on a 96-well white-walled plate, infected or transfected with VLPs, and after incubation the supernatant was removed, and the cells were gently washed with PBS. Next, 25 μL of the nLuc reagent (1:50 in manufacturer’s buffer) was added to the cells, and they were incubated for 3 min, after which the signal was read on a BMG Clariostar Fluorescent plate reader. To measure secreted nLuc, 20 μL of the supernatant was treated with or without 2 μg/mL of PK for 10 min at RT, and then 20 μL of nLuc reagent was added, incubated for 3 min, and the signal was measured.

### 2.7. Virus Growth, Purification, and nanoLuciferase Measurement

SARS-Related Coronavirus 2 Isolate USA-WA1/2020 was grown in VeroE6-TMPRSS2 to passage 3, titrated by plaque assay in VeroE6-TMPRSS2 with 0.75% Carboxymethyl cellulose (Sigma-C4888), overlaid for 72 h, and stained with 1% crystal violet. For experiments, the MOI was calculated and the virus was added to HEK293T-Ace2-TMPRSS2 cells (a gift from Jennifer Doudna), expressing nanoLuciferase for the indicated times and treatments, and the samples were again titrated by plaque assay, and the RNA was extracted with TRIZOL. To measure the secreted nLuc, the supernatant was centrifuged at 10,000× *g* for 20 min and treated with either 1% triton, protease K, or nothing, and then incubated with the nLuc reagent for 3 min, after which the luminescence was measured on a Promega GloMax Discover plate reader.

The Dengue virus (16681) and ZIKV-PRV were both passaged on C636 cells and titrated on BHK21 cells following established protocols [28]. OC43 and NL63 (ZeptoMetrix, Buffalo, NY, USA) were both passaged on VeroE6-TMPRSS2 cells up to passage three and titrated by plaque assay in VeroE6-TMPRSS2 with a 0.75% Carboxymethyl cellulose (Sigma-C4888) overlay for 8–10 days and stained with 1% crystal violet. HEK293T-Ace2-TMPRSS2 or HEK-293T cells expressing nanoLuciferase were infected with OC43, NL63, DENV2, or ZIKV at MOI = 0.5. Supernatants were collected at 24 and 48 h and filtered through a 0.45 μM PES filter. A total of 20 μL of the supernatants was added to a 96-well white walled plate and treated with PK for 10 min and incubated with the nLuc reagent, and the luminescence was measured on the BMG Clariostar Fluorescent plate reader.

### 2.8. Protease Protection Assay

For the protease protection assays, 20 μL of concentrated VLP or virus or mock supernatant were incubated with or without 1% triton for 30 min at RT, then treated with or without 2 mg/mL protease K (ThermoFischer Scientific, Waltham, MA, USA) for 10 min at RT. The nLuc, renilla, or firefly reagents were added for 3 min, and the luminescence was measured on a BMG Clariostar Fluorescent plate reader (VLPs, NL63 and OC43) or a Promega GloMax Discover plate reader (SARS-CoV-2).

### 2.9. Spike Mini-Binder Pull-Down

To prepare the spike-binder for the VLP and virus pull down, we added a C terminal cysteine to LCB1, which has no other cysteines, and an N terminal 6xHis tag followed by a TEV cleavage site. We purified the protein similar to established protocols [29]. Briefly, the plasmid was expressed in Rosetta BL21 *E. coli* (SigmaAldrich), protein production was induced with IPTG, and the cells were grown overnight at 18 C. The lysate was prepared and loaded onto a nickel column, and washed and eluted with 400 mM imidazole. We then cleaved the 6xHis tag with TEV and loaded the protein onto a Superdex75 size-exclusion chromatography column (GE Healthcare Life Sciences). Fractions containing the binder were identified using SDS-PAGE, pooled, and buffer exchanged through a 3 kDa cutoff amicon filter before snap freezing.

The protein was biotinylated with a maleimide reaction, where the LCB1 was diluted in PBS to 100 μM and incubated with 2 mM Pierce Maleimide-biotin (ThermoScientific Scientific, Waltham, MA, USA) and 0.5 M TCEP for 2 h at room temperature. The biotinylated protein was separated from the un-bound label with a PD-10 desalting column (GE-Healthcare). The protein concentration was measured with BCA, and the aliquots were stored at −80 °C. The biotinylated protein was conjugated to Pierce streptavidin magnetic beads (ThermoFischer Scientific, Waltham, MA, USA) by incubating 100 μL of a 1 μM biotinylated-binder with 30 μL of beads for 1 h at room temperature, and then washed and stored in PBS.

The prepared beads or mock beads with no binder were blocked with 3% BSA for 1 h at room temperature, washed in PBS, and incubated with 1 mL of virus or VLP supernatant overnight at 4 °C with gentle rotation. The beads were separated from the flow-through on a magnetic rack and washed three times in PBS. The beads were then treated with or without 2 mg/mL PK to remove un-packaged nLuc, and incubated with the nLuc reagent for three minutes. The beads were separated on a magnetic rack, the eluate was transferred to a white 96-well plate, and the luminescence was measured on a BMG Clariostar Fluorescent plate reader (VLPs) or a Promega GloMax Discover plate reader (virus).

### 2.10. siRNA Treatments

The siRNAs used were synthesized by Millipore Sigma for the RdRP siRNA [30], and the negative control #1 from ThermoFisher was used as a control. For the siRNA treatment, HEK293T-Ace2-TMPRSS2 cells were transfected using Lipofectamine 2000 (Thermo Fischer Scientific, Waltham, MA, USA) with 100 μM of siRNAs and 0.8 μg of the nLuc plasmid, and incubated for 24 hr. The cells were then infected with SARS-CoV-2 MOI = 0.5. The cells and the supernatant were collected for nLuc reading, plaque assay, and RNA extraction.

### 2.11. Inhibitor Treatments

The inhibitors were dissolved in DMSO and further diluted into complete media at 0.1% DMSO and added to the cells. Optimal concentrations were determined with cytotoxicity measurements by adding resazurin [Alamar blue (ThermoFisher Scientific, Waltham, MA, USA)] diluted to 1× in DMEM according to the manufacturer’s protocol and incubated on cells for 1–2 hr. Fluorescence was measured on a BMG Clariostar Fluorescent plate reader with excitation at 560 nM and emission at 590 nM. Cells expressing nLuc were infected with the indicated virus for 1 h at 37 °C, after which the virus inoculum was removed, and DMEM with 2% FBS and the indicated inhibitor was overlaid on the cells. After 48 h, the virus supernatant was collected, treated with PK, and nLuc was measured.

## 3. Results

### 3.1. Development of SARS-CoV-2 Virus-Like Particles Bearing a Reporter mRNA

**Figure 1:**
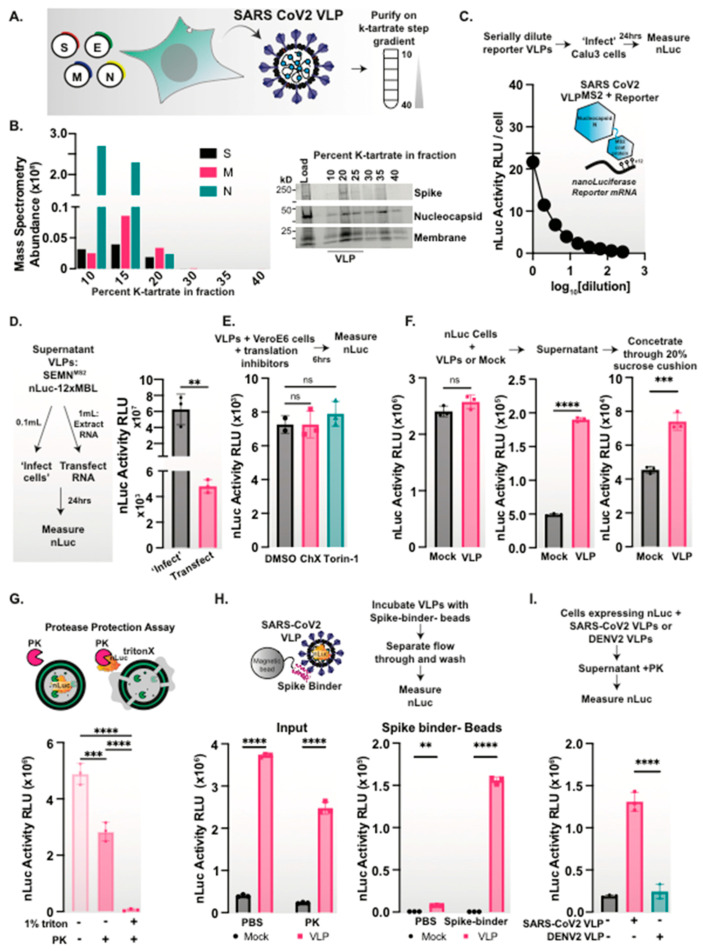
The nanoLuciferase protein is packaged and secreted in SARS-CoV-2 VLPs. (**A**) Schematic of our SARS-CoV-2 VLP system. The SARS-CoV-2 structural proteins: Spike (S), Envelope (E), Membrane (M) and Nucleocapsid (N) proteins are co-expressed in cells, and together form particles that are secreted that we can purify and characterize. (**B**) VLP proteins are co-secreted from cells. The supernatant VLPs were collected, concentrated, and run on a density step-gradient. Fractions were collected, and SARS-CoV-2 structural proteins were identified by mass spectrometry and western blot. (**C**) VLPs with an nLuc reporter RNA are delivered to naïve cells. Reporter VLPs with N protein genetically fused to the MS2 capsid protein and nanoLuciferase (nLuc) with 12 copies of the MS2 binding loops (MBL) were serially diluted and added to naïve indicated cells and incubated for 24 h, after which the nLuc signal was measured. (**D**) VLPs delivered directly to cells have a higher nLuc signal than transfected RNA. A schematic of the ‘infection’ and transfection approaches to deliver reporter VLPs to new cells. VLPs with the nLuc-12x construct were produced, and RNA was extracted from the supernatant. The supernatant was added directly to naïve cells, while other naïve cells were transfected with the RNA. The nLuc signal was read after 24 h of incubation. (**E**) The nLuc signal in naïve cells is not dependent on cellular translation. Reporter VLPs were added to naïve cells and treated with cycloheximide or torin-1 for 6 h to prevent any translation of the reporter message. We see no difference in the nLuc signal that would indicate that the signal does not arise from the nascent translation of the reporter RNA. (**F**) The nLuc protein is co-secreted with VLPs. The nLuc reporter along with the indicated VLP proteins were transfected into cells and incubated for 48 h. The supernatant was removed, filtered, and the nLuc signal was measured in both the cells and directly in the supernatant. The supernatant was concentrated through a 20% sucrose cushion, and the nLuc signal was measured in the pellet. (**G**) A schematic of a protease protection assay. The nLuc is secreted in a PK-protected, triton-sensitive vesicle. The VLPs were produced with nLuc and concentrated through a 20% sucrose cushion. The purified VLP sample was then left untreated or treated with 1% triton followed by protease K (PK). The samples treated with only PK were protected, while the nLuc signal was removed by PK in the triton disrupted samples, indicating that a majority of the nLuc was packaged in a vesicle. (**H**) VLPs package nLuc. The nLuc activity was measured in a supernatant from mock and VLP expressing cells, which were then incubated with magnetic beads conjugated to the spike-binder, LCB1. The beads were washed and the nLuc signal on the beads was measured. (**I**) nLuc is not packaged into DENV2 VLPs. HEK293T cells were transfected with nLuc and GFP or SARS-CoV-2 or DENV2 VLP proteins. The supernatant was collected after 48 h, filtered, treated with PK, and the nLuc signal was measured. A Student’s T-test, one-way or two-way ANOVA with multiple comparisons tests were performed: ns = not significant, ** = *p* < 0.01, *** *p* < 0.001, **** *p* < 0.0001; *n* = 3.

Producing SARS-CoV-2 VLPs would be ideal to understand viral production and entry, as they mirror an authentic virus significantly better than pseudovirus platforms only containing the displayed lentivirus spike protein. SARS-CoV-2 VLP production by co-expression of the four structural proteins was originally designed for SARS-CoV1 VLP production [31,32,33,34]. Similar to previous reports [12,13,14,15,16,17,18,19], we produced SARS-CoV-2 VLPs by co-expressing the four canonical structural proteins of SARS-CoV-2: spike (S), envelope (E), membrane (M), and nucleocapsid (N) in HEK293T cells. VLPs collected from the supernatant were purified by sedimentation through sucrose cushions, followed by analysis on potassium tartrate density gradients (Figure 1A). The co-migration of structural S, M and N proteins at regions of the density step gradient where the WT virus sediments was observed both by immunoblotting and mass spectrometry were consistent with the formation of VLPs (Figure 1B). The E protein was hard to detect, likely because it is very small (11 kDa), hydrophobic, and is expected to be packaged at very low levels [14,35,36]. These analyses indicate that VLPs were assembled and secreted from producer cells.

We next tested a recently reported VLP system that incorporates a reporter encoding mRNA using a SARS-CoV-2 viral packaging signal system [12]. Upon the addition of these VLPs to Ace2-expressing cells, we observed the encoded Firefly Luciferase (FLuc) yielding activity in the recipient cells, but the very low signal level limits the dynamic range of measurements (Supplemental Figure S1A). Therefore, we set out to build an alternative reporter system for SARS-CoV-2 VLPs. We reasoned that the bacteriophage capsid MS2-RNA binding interaction, which has a well validated specificity and a higher affinity, could enhance the ability of VLPs to incorporate more copies of reporter mRNA into the particle, yielding an improved signal to background ratio upon delivery to recipient cells. As reporters, we chose GFP and nanoLuciferase (nLuc). Similar to FLuc, nLuc has a facile and sensitive luminescence assay [37]. Our VLP design consisted of the four SARS-CoV-2 structural proteins, where the N protein carried a C-terminal fusion to the MS2 bacteriophage capsid protein (N^MS2^ [38]), as well as a reporter gene containing 12 copies of the improved MS2 bacteriophage packaging signal MBVSV6 [25] (Figure 1C and Figure S1B). In this design, the MS2 capsid domain in N^MS2^ should recruit the mRNA carrying the MS2-binding loops (herein MBL) to yield a VLP carrying the encoded reporter in the viral particle (herein VLP^MS2^). To assess the presence of the GFP reporter mRNA in VLP^MS2^, we collected the secreted VLPs and treated them with RNase A to digest any mRNA that was not encapsulated. Following RNase A inactivation, we extracted the RNase-resistant RNA using Trizol and examined the levels of reporter RNAs by qRT-PCR. We only detected the reporter mRNAs in the supernatant of VLP^MS2^, i.e., those generated using N^MS2^, but not when the VLPs were generated by N without the MS2 protein domain. This suggests that N^MS2^ recruits the MS2-tagged mRNA into VLP^MS2^ (Supplemental Figure S1E). Importantly, by using primers that amplify the entire expected RNA construct: ~1400 bp of the nLuc and 12xMBL loops, we confirmed the VLP^MS2^ particles package and secreted the full-length reporter mRNA (Supplemental Figure S1F). We amplified RNA from purified VLPs with a longer extension time to make cDNA, followed by agarose gel analysis. This revealed the VLPs package full-length RNA, as expected for mRNA that are competent as a reporter (Supplemental Figure S1F).

Finally, we tested the ability of our reporter VLP^MS2^ bearing nLuc to pseudo-infect a panel of ACE2 expressing cells. The VLPs were serially diluted and added to naïve cells and allowed to attach for 1 h, then the inoculum was washed and fresh media was added. After a 24 h incubation, cells were lysed and nLuc activity was quantified. For all of the cell types, we observed a robust dose-dependent nLuc signal, indicating the utility of the VLP^MS2^ reporter system (Figure 1C and Figure S1C).

### 3.2. The nanoLuciferase Protein Is Packaged and Secreted in SARS-CoV-2 VLPs

To determine the efficiency of the VLP^MS2-^ based delivery of RNA, we compared nLuc activity resulting from either direct ‘infection’ with the nLuc-VLP^MS2^ compared to direct transfection or the Trizol-extracted mRNA from nLuc-VLP^MS2^. A fraction (0.1 mL) of the filtered supernatant of the cells producing nLuc-VLP^MS2^ was directly added to naïve HEK293T-Ace2/TMPRSS2 cells, while 1 mL of the same supernatant was used to extract mRNA, which was transfected into naïve cells (Figure 1D). After 24 h, we measured the nLuc signal in the recipient cells. Both approaches led to nLuc expression in naïve cells (Figure 1D). However, the nLuc signal from the direct ‘infection’ was significantly higher than that obtained by the transfection of RNA. To ensure that the nLuc activity in cells “infected” with nLuc-VLP^MS2^ arises from reporter mRNA translation, we added protein synthesis inhibitors, cycloheximide or torin-1, to nLuc-VLP^MS2-^ infected cells. Surprisingly, translation inhibition did not diminish nLuc expression upon the VLP addition (Figure 1E). Therefore, we considered whether the nLuc-VLP^MS2^ contained the nLuc protein in addition to the reporter mRNA, and whether this contributed substantially to the nLuc signal from the VLP “infection”. Indeed, both the filtered supernatant from cells expressing nLuc-VLP^MS2^ or VLPs purified through sucrose cushion sedimentation both contained robust nLuc activity (Figure 1F).

To assess whether nLuc copurifies with the VLPs, we subjected sucrose-cushion purified VLP-nLuc to fractionation on a potassium tartrate density step-gradient and assessed SARS-CoV-2 structural proteins and nLuc activity in each fraction. Indeed, nLuc activity was found in similar density fractions as the VLP proteins (Supplemental Figure S1G). We then used a protease protection assay to determine whether nLuc was contained within a lipid enveloped structure such as that of SARS-CoV-2 VLPs. Indeed, most of the nLuc signal was protected from the Proteinase K (PK) digestion unless the PK treatment was carried out after treatment with 1% triton to disrupt the envelope (Figure 1G). Taken together, these findings indicate that the nLuc protein is packaged within SARS-CoV-2 VLPs.

Since we observed low levels of the nLuc signal in our mock samples (Figure 1F), we considered whether nLuc is packaged into VLPs or whether it can be secreted in exosome-like vesicles in a process upregulated by the VLP proteins. To test whether nLuc is packaged inside the VLPs, we developed a SARS-CoV-2 spike-specific affinity purification protocol. To this end, we generated a biotinylated form of the spike-binding protein LCB1, [29] and conjugated it to streptavidin magnetic beads (herein LCB1-beads). Incubating the supernatant of cells expressing nLuc or nLuc-VLPs with LCB1-beads confirmed that the nLuc is packaged directly inside the spike containing VLPs (Figure 1H). First, we observed significantly higher PK-resistant nLuc activity in the supernatant of nLuc-VLP expressing cells (Figure 1H). Secondly, we observed strong nLuc activity on the LCB1-beads incubated with nLuc-VLPs compared to mock (Figure 1H).

To determine if other VLP systems also promote nLuc secretion, we compared the SARS-CoV-2 VLPs system to a DENV2 VLPs system in which the DENV2 structural proteins Capsid, prM, and Envelope proteins are co-expressed in a single plasmid [24]. Cells expressing nLuc were transfected with either the SARS-CoV-2 VLP or the DENV2 VLP plasmids, and the VLPs were collected, filtered, and treated with PK to remove any soluble nLuc protein. We then measured the nLuc activity in the treated VLP. We found a robust secretion of nLuc with SARS-CoV-2 VLPs but not with DENV2 VLPs (Figure 1I), suggesting a SARS-CoV-2 specific effect.

### 3.3. Coronaviruses but Not Flaviviruses Package nanoLuciferase Reporters in Their Viral Particles

**Figure 2:**
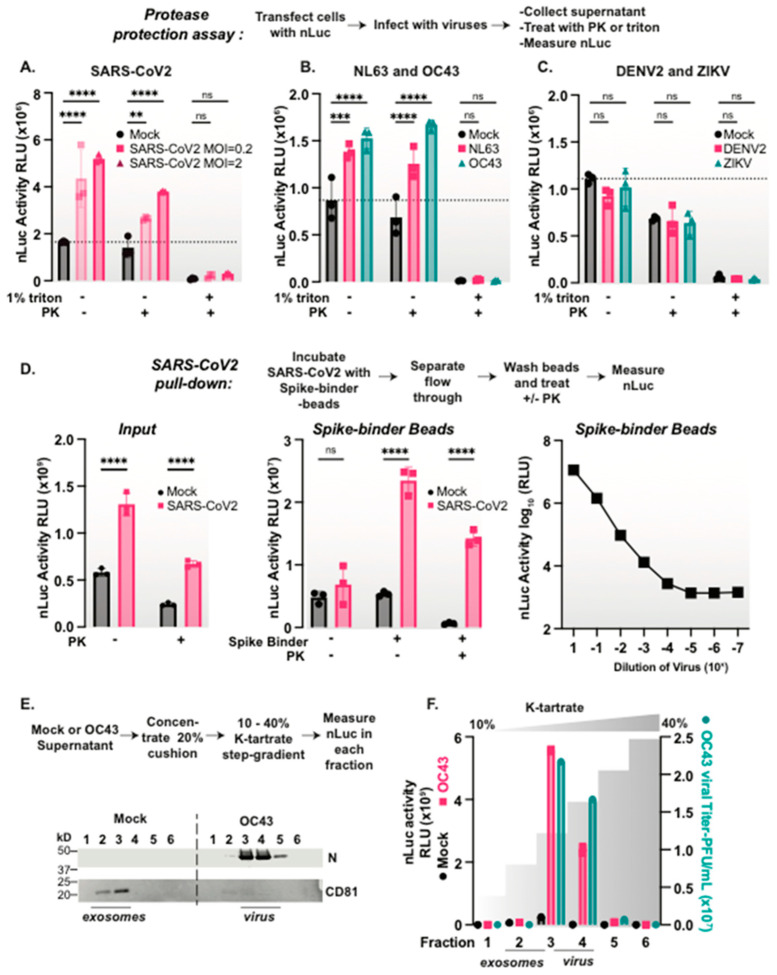
SARS-CoV-2 packages nanoLuciferase inside a viral particle. (**A**–**C**) Select coronaviruses package nLuc, while flaviviruses do not. Protease protection assay: nLuc expressing cells were infected with (**A**) SARS-CoV-2, (**B**) NL63, OC43, (**C**) DENV2, ZIKV or mock for 48 h, after which the supernatant was collected and treated with or without 1% triton followed by PK before measuring the nLuc signal. (**D**) nLuc is packaged inside SARS-CoV-2 viral particles. nLuc expressing cells were infected with SARS-CoV-2 or mock infected for 48 hrs, after which the supernatant was collected and the input nLuc signal was measured. The samples were then incubated with streptavidin magnetic beads conjugated to the spike-binder, or the beads alone overnight at 4 C. The flow-through was removed, the beads were washed in PBS and treated with or without PK, and the nLuc signal was measured. Additionally, the virus input sample was serially diluted 10-fold and incubated with spike-binder-conjugated beads, washed, and the nLuc signal was measured. (**E**,**F**) nLuc co-purifies with OC43 virions. The OC43 virus or mock supernatant was collected from nLuc expressing cells, concentrated through a sucrose cushion and purified on a k-tartrate density step gradient. Each fraction was analyzed for (**E**) N and CD81 protein signals, and (**F**) nLuc activity and infectious virus titer. Two-way ANOVA with multiple comparisons tests were performed: ns = not significant, ** = *p* < 0.01, *** *p* < 0.001, **** *p* < 0.0001; *n* = 3.

We next asked whether the SARS-CoV-2 virion can package nLuc similarly to the VLPs. We infected nLuc expressing HEK293T-Ace2TMPRSS2 cells (Figure 2A) or A549-Ace2 cells (Supplemental Figure S2B) with SARS-CoV-2 using two different multiplicities of infections (MOIs). After 48 hrs, we compared the nLuc activity in the supernatants and the total cell extracts in the presence and absence of virus infection. While nLuc was expressed in all cells, infection with SARS-CoV-2 increased the nLuc signal in the supernatant (Supplemental Figure S2B and Figure 2A). As observed for the VLPs, the nLuc secreted from cells upon SARS-CoV-2 infection was resistant to PK digestion, but was rendered PK sensitive upon treatment with 1% triton (Figure 2A). These findings suggest that SARS-CoV-2 can encapsidate endogenously the expressed nLuc protein.

To assess the generality of our findings, we examined whether other enveloped viruses can promote the secretion of the PK-resistant nLuc protein upon the infection of nLuc expressing cells. First, we tested two common cold coronaviruses: NL63 and OC43. We infected nLuc expressing cells at MOI = 0.5, collected the supernatant, and following a PK protease protection assay we measured the nLuc activity. Indeed, both NL63 and OC43 induced the secretion of protease resistant nLuc that was rendered PK sensitive upon treatment with 1% triton (Figure 2B). This suggests multiple coronaviruses package nLuc in a protease protected, triton-sensitive structure upon viral infection.

We next tested whether flaviviruses also promote nLuc secretion. We infected nLuc expressing cells with either DENV2 strain 16681 [39] or ZIKV (PRVABC59) [40] at MOI = 0.5, collected the supernatant at 48 h, and examined the levels of PK-resistant nLuc activity. Unlike what is observed with coronaviruses, there was no increase in the nLuc signal with either DENV2 nor ZIKV compared to mock infected cells (Figure 2C). Thus, nLuc secretion is not a common feature of all enveloped viral infections, suggesting that it is a feature of coronaviruses, perhaps resulting from their very large virions.

### 3.4. nLuc Is Packaged in SARS-CoV-2 Virus Particles

To directly test whether nLuc is packaged inside viral particles, we resorted to the affinity purification of viral particles with the spike binding LCB1-beads. Following the SARS-CoV-2 infection of nLuc expressing cells, we observed increased PK-resistant nLuc activity in the supernatant of viral infected cells (Figure 2D). We next incubated the PK treated supernatants of virus-infected or mock cells with LCB1 or control beads. These experiments confirmed the presence of PK-resistant nLuc activity directly inside spike-containing viral particles (Figure 2D). A serial dilution of the virus supernatant prior to the affinity purification on LCB1-beads revealed nLuc bound beads in a dose-dependent manner (Figure 2D).

We wanted to determine if nLuc is also packaged directly inside the viral particle of another coronavirus, OC43. Since we did not have an OC43 spike-specific binder, we chose a density gradient approach to purify the viral particles. Supernatants from mock or OC43-infected nLuc expressing cells were concentrated through a sucrose cushion, and the particle containing pellet was loaded on to a potassium tartrate density step gradient. We then examined each fraction of the gradient to detect the nLuc activity and any infectious virus. We also examined the elution profile of exosomes by immunoblot for CD81 and viral particles by immunoblot for nucleocapsid proteins. The virus-infected samples had significantly more nLuc activity than those of the mock infected cells. Furthermore, the fractions with the highest nLuc activity also had the highest level of infectious virus and nucleocapsid protein (Figure 2E,F). Fractions enriched in exosomes did not comigrate in the gradient with infectious viral particles, indicating that the gradient separates the exosomes from the virus. These experiments indicate that nLuc is packaged into the OC43 viral particles. Together, these findings demonstrate that nLuc is directly packaged into coronavirus viral particles. Since nLuc also appears to be secreted at low levels in exosomes during normal cellular processes, our data raise the interconnectedness of viral assembly and cellular secretory pathways.

### 3.5. Size Constrains Non-Specific Protein Packaging into Coronavirus Particles

**Figure 3:**
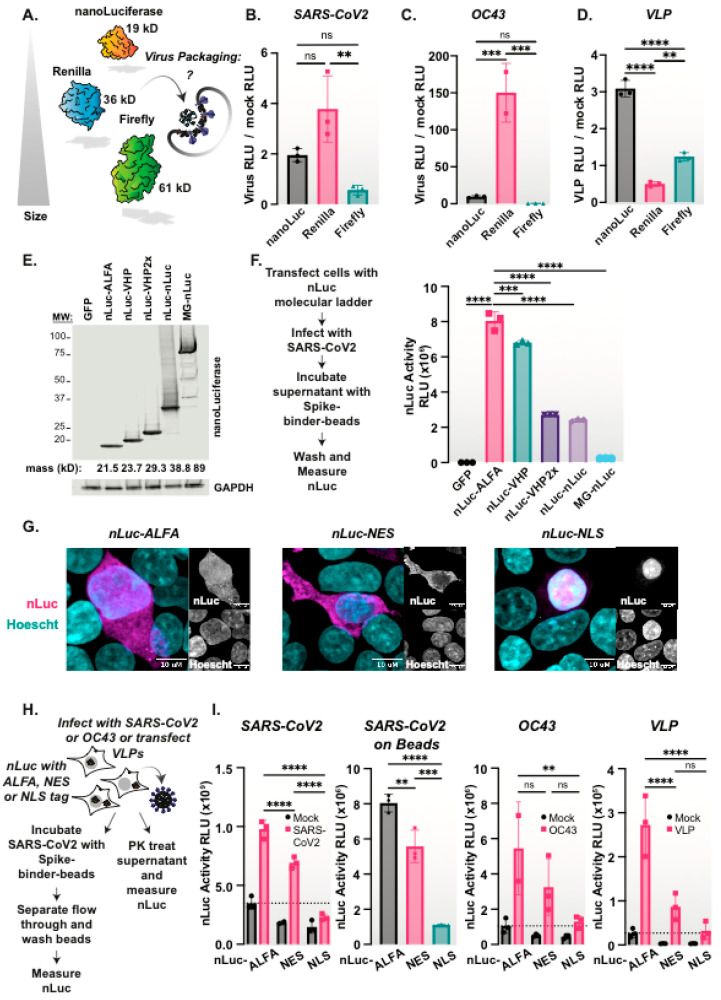
SARS-CoV-2 virions exhibit size dependence in their non-specific cargo packaging. (**A**) A schematic of the nLuc, renilla, and firefly luciferases size differences and packaging into budding virions. (**B**–**D**) SARS-CoV-2 and OC43 virions package nano- and renilla-, but not firefly luciferase, while VLPs only package nLuc. Cells expressing nLuc, renilla or firefly luciferases were infected with (**B**) SARS-CoV-2, (**C**) OC43, (**D**) co-expressed with SARS-CoV-2 VLPs for 48 h. The supernatant was collected, treated with PK, and the luciferase activity was measured with the corresponding reagent. (**E**) Modification of nLuc with various size tags. nLuc constructs were expressed in HEK293T-Ace2-TMPRSS2 cells, which were processed for western blotting, demonstrating size differences. (**F**) SARS-CoV-2 preferentially packages smaller nLuc species. The five nLuc constructs or a GFP control were transfected in cells and subsequently infected with SARS-CoV-2, MOI = 0.5 for 48 h. The supernatant was collected, incubated with the spike-binder beads overnight at 4 C, washed, and the nLuc activity on the beads was measured. (**G**) Localization of nLuc with -ALFA, -NES or -NLS tags. nLuc localization constructs were expressed in HEK293T cells which were fixed in 4% PFA and processed for the immunofluorescence of nLuc. (**H**) Schematic of a virion nLuc packaging assay depending on cellular sub-localization. (**I**) Nuclear localized nLuc is not packaged into SARS-CoV-2, OC43 or SARS-CoV-2 VLPs. Cells expressing nLuc with -ALFA, -NES or -NLS tags were infected with SARS-CoV-2, OC43, or co-expressed with SARS-CoV-2 VLPs for 48 h. The SARS-CoV-2 supernatant and mock control was collected, treated with PK, and the nLuc activity was measured, or the virus was directly incubated with the spike-binder beads, and the nLuc activity was measured after washing the beads. The OC43 or VLP supernatants and mock controls were collected, treated with PK, and the nLuc activity was measured. One or two-way ANOVA with multiple comparisons tests were performed: ns = not significant, ** = *p* < 0.01, *** *p* < 0.001, **** *p* < 0.0001; *n* = 3.

We next explored the plasticity of the non-specific incorporation of proteins expressed in the infected host cell into coronavirus particles. Since nLuc is a small 19 kDa protein, we also tested the incorporation of two additional luminescent proteins, the 36 kDa Renilla luciferase and the 61 kDa Firefly luciferase [37]. Cells expressing each of the three luciferase proteins were infected with either SARS-CoV-2 or OC43. In addition, we expressed the VLP-generating SARS-CoV-2 proteins in these cells to assess luciferase incorporation (Figure 3A). In all cases, VLP expression or viral infection did not alter the levels of luciferase activity in the total cell extracts (Supplemental Figure S3). We then collected the supernatants of infection or VLP expression and processed them as described above, using the PK protease protection assay to quantify the packaged and secreted luciferase across different samples. Both SARS-CoV-2 and OC43 viral particles contained nLuc and Renilla luciferase, but not the larger Firefly luciferase (Figure 3B,C and Figure S3). In contrast, SARS-CoV-2 VLPs only packaged nLuc, and did not contain either the Renilla or Firefly luciferase compared to mock cells (Figure 3D). We hypothesize that the differential virion packaging of host cell luciferase proteins of distinct size is constrained by its capacity to fit extra cargo into the viral particles. This idea is further supported by the inability of VLPs to package Renilla luciferase, which is packaged by virions. VLPs migrate slightly higher on a density gradient compared to virions (compare Figure 1B and Figure 2E), suggesting that VLPs are less dense and perhaps smaller than virions. Alternatively, VLPs may lack the viral packaging machinery that facilitates non-specific packaging of all but very small proteins.

To further test the constraints of non-specific reporter protein packaging into virions, we generated a molecular ladder of nLuc constructs ranging in size from 21 kDa to 89 kDa. We generated fusions between nLuc and a small ALFA tag, 21.5 kDa [41], either one or two copies of the villin “headpiece” (VHP), 23.7 kDa and 29.3 kDa, respectively [26], two copies of nLuc in tandem, 38.8 kDa, and MBP-GFP, 89 kDa. The expression of these constructs in cells followed by the nLuc immunoblot revealed the efficient expression at their expected molecular weight (Figure 3E). Following infection with SARS-CoV-2, we collected the supernatant, isolated viral particles by affinity purification with the spike-binder LCB1-beads, and measured the nLuc activity. Notably, as the size of the host-cell expressed nLuc protein increased, there was less nLuc activity in the virions (Figure 3F). These data suggest that the size of the host-cell expressed proteins constrains their non-specific packaging into coronavirus VLPs or virus particles.

### 3.6. Subcellular Location Constrains nLuc Packaging into Coronaviruses

Similar to other positive strand RNA viruses, coronaviruses assemble on cytoplasmic membranes [8,42]. Therefore, we reasoned that nLuc would likely be non-specifically recruited from the cytoplasm into the virion. To test this, we C-terminally fused nLuc to either an NLS or NES localization signal or a similarly sized ALFA tag and expressed these variants in HEK293T cells. nLuc immunostaining and imaging by confocal microscopy revealed that ALFA-tagged nLuc was distributed throughout the cell, as was expected for such a small protein (Figure 3G), while nLuc-NES was exclusively cytoplasmic and nLuc-NLS was nuclear (Figure 3G). Next, we infected the nLuc-variant expressing cells with SARS-CoV-2, collected the supernatant, treated it with PK, and measured the nLuc activity. We also incubated the supernatant with spike-binder LCB1-beads to measure the virion-contained nLuc activity (Figure 3H). We observed a significant increase in PK-resistant nLuc activity in the supernatants and the LCB1-bound virions of SARS-CoV-2 infected cells that expressed either nLuc-ALFA or nLuc-NES. In contrast, infected cells expressing nuclear-restricted nLuc-NLS did not produce PK-resistant nLuc in the supernatant or incorporate it into viral particles (Figure 3I). Similar results were observed for a second coronavirus, OC43, as well as for SARS-CoV-2 VLPs, namely that the ALFA-tagged and NES-tagged nLuc were incorporated into virions or VLPs, while the nLuc-NLS was not (Figure 3I). Therefore, nLuc must localize to the cytoplasm, likely near the sites of particle assembly, in order to be non-specifically packaged into budding virions.

### 3.7. nanoLuciferase Activity Reports on SARS-CoV-2 Replication Levels

**Figure 4:**
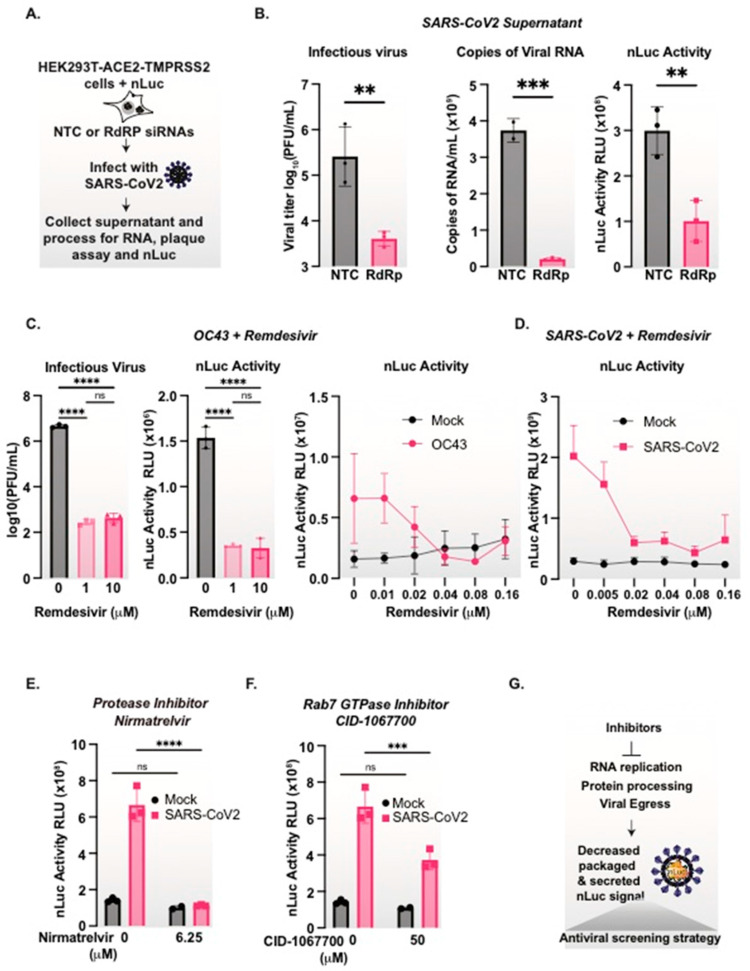
SARS-CoV-2 associated secreted nanoLuciferase mirrors viral particle secretion. (**A**) Schematic of cells transfected with nLuc and siRNAs with viral infection. (**B**) Knockdown of SARS-CoV-2 RdRp reduces infectious virus and nLuc packaged particle formation. HEK293T-ACE2/TMPRSS2 cells were treated with siRNAs targeting the RdRp or a non-targeting control. The cells were subsequently infected with SARS-CoV-2, MOI = 0.5 for 48 h. The supernatant was collected and processed for viral genome equivalents, infectious virus via a plaque assay, and nLuc signals. (**C**) The inhibition of OC43 replication reduces infectious virus and secreted nLuc in a dose-dependent manner. nLuc expressing cells were infected with OC43 MOI = 0.5 and treated with the indicated concentrations of Remdesivir. The supernatant was collected after 48 h and titrated via a plaque assay or treated with PK, and nLuc activity was measured. (**D**) The inhibition of SARS-CoV-2 replication reduces secreted nLuc in a dose-dependent manner. nLuc expressing cells were infected with SARS-CoV-2 and treated with the indicated concentrations of Remdesivir for 48 h, after which the supernatant was collected, treated with PK, and nluc was measured. (**E**) The protease inhibitor Nirmatrelvir reduces the secreted SARS-CoV-2 nLuc particles. nLuc expressing cells were infected with SARS-CoV-2 and treated with the indicated concentration of Nirmatrelvir for 48 h, after which the supernatant was collected, treated with PK, and the nluc activity was measured. (**F**) The Rab7 GTPase inhibitor CID-1067700 reduces secreted SARS-CoV-2 nLuc particles. nLuc expressing cells were infected with SARS-CoV-2 and treated with the indicated concentration of CID-1067700 for 48 h, after which the supernatant was collected, treated with PK, and the nluc activity was measured. (**G**) Packaged and secreted nLuc mirrors coronavirus particle production and can be utilized as an antiviral screening assay. One- or two-way ANOVAs with multiple comparison tests were performed: ns = not significant, ** = *p* < 0.01, *** *p* < 0.001, **** *p* < 0.0001; *n* = 3.

We next assessed whether we can employ the PK-resistant nLuc activity in the supernatant of SARS-CoV-2 infected cells as a readout for virus production. To this end, we compared the effect of treatments or drugs that inhibit viral replication on both nLuc activity and viral RNA copies in the supernatant as well as the infectious titers. First, we carried out the siRNA knockdown of the viral RNA-dependent RNA polymerase (RdRP), an intervention that reduces viral replication. Cells expressing nLuc were treated with either non-targeting siRNA or an siRNA targeting the SARS-CoV-2 RdRP [30], and were infected with SARS-CoV-2 at MOI = 0.5. After 48 h, the supernatant was collected and viral RNA was measured via qRT-PCR, infectious virus titers were measured via a plaque assay, and nLuc activity was measured after PK-treatment (Figure 4A). As expected, RdRp knockdown led to a significant decrease in secreted viral RNA and infectious virus titers (Figure 4B). Of note, the siRNA of RdRP also led to a significant decrease in secreted nLuc (Figure 4B) that mirrored the decrease in viral RNA and infectious virus titers (Figure 4B).

To assess whether nLuc can be used as a rapid screening reporter for inhibitors of coronavirus replication, we used the nLuc secretion assay to examine the effect of broad-spectrum antiviral Remdesivir [43,44] on the replication of either OC43 or SARS-CoV-2 (Figure 4C,D). First, nLuc-expressing cells infected with OC43 were treated with two concentrations of Remdesivir for 48 h. We observed similar drug-dependent reductions in viral titers and PK-resistant nLuc activity, indicating that the nLuc signal mirrors the viral production (Figure 4C). We then tested whether the nLuc signal is sensitive to the dose-dependent inhibition of viral replication. OC43 infected (Figure 4C) and SARS-CoV-2 infected (Figure 4D) nLuc expressing cells were treated with a series of two-fold dilutions of Remdesivir for 48 h. For both coronaviruses, we observed that Remdesivir led to a dose-dependent decrease in the secreted PK protected nLuc signal.

The generality of the nLuc assay as a reporter of viral replication was extended to two additional anti-coronavirus compounds working through two completely different mechanisms. We first tested Nirmatrelvir, the active compound in Paxlovid targeting SARS-CoV-2-chymotrypsin-like cysteine protease enzyme (M^pro^) [45,46] (Figure 4E). Finally, we tested a host-directed compound that preferentially inhibits coronavirus secretion, namely the Rab7 GTPase inhibitor CID-1067700 [47] (Figure 4E). Treatment with either antiviral compound led to a decrease in the viral-induced secreted PK-protected nLuc signal. Therefore, we conclude that nLuc packaging and secretion by coronaviruses can be used as a rapid, versatile and sensitive assay to measure coronavirus replication and release in antiviral screening assays, without the need for cloning or engineering (Figure 4G).

## 4. Discussion

In this study, we have demonstrated a novel and versatile method to rapidly measure secreted SARS-CoV-2 viral particles that mirror secreted viral RNA and infectious virus production and that can be applied broadly to other coronaviruses. We set out to produce a novel reporter RNA construct for VLP entry assays, but we found that the small protein nanoLuciferase is packaged within both SARS-CoV-2 VLPs as well as infectious virions. This led us to discover a novel phenomenon of SARS-CoV-2 viral particle assembly, namely the non-specific encaspidation of at least some cytoplasmic proteins expressed in the host cell. We demonstrated that this feature can be exploited as a tool to measure coronavirus particle secretion.

While our engineered VLP particles did package full length reporter mRNA that can be translated in recipient cells, its pseudo-infectivity-based activity is very low. Few other SARS-CoV-2 VLP systems have demonstrated spike-ACE2 dependent pseudo-infectivity, but these systems are also very inefficient, with only 3–5% of cells becoming ‘infected’ with VLPs [12,13]. Therefore, proper SARS-CoV-2 VLP assembly is an inefficient process in the absence of viral infection.

Surprisingly, when expressed in the host cell, the nLuc protein itself is secreted in a PK protected, triton sensitive particle during both VLP production or infection with a panel of coronaviruses: OC43, NL64, and SARS-CoV-2. Interestingly, in uninfected cells, we observe a very low level of protease protected nLuc secretion, which might correspond to exosomes. However, our data demonstrates that nLuc is packaged directly inside viral particles when expressed in the host cytoplasm.

In addition to providing a sensitive and facile assay, it is of interest to better understand the mechanism by which nLuc is encapsidated in coronavirus virions. We hypothesize that the mechanism is non-specific and involves the passive diffusion of the small nLuc into budding virus assembly sites. Indeed, nLuc is not incorporated into the virions of flaviviruses DENV2 and ZIKV, which produce smaller viral particles of approximately 50 nM in diameter by budding into the lumen of ER membranes [48]. The much larger SARS-CoV-2 virions, approximately 100–150 nM in diameter [7,8], may have the cargo-carrying capacity to incorporate small cytosolic proteins. It will be of interest to determine what additional host proteins are similarly encapsidated into coronavirus virions, and SARS-CoV-2 in particular, and whether some specific host proteins play a role in subsequent virus infection. Of note, a variety of host proteins have been reported to associate with purified virions of select coronaviruses [49,50,51]. Many of these host proteins bind specifically to viral structural proteins, while others have no known interactions, raising the possibility they are also packaged non-specifically, as we observed for nLuc. Cryo-EM structures of intact virions [8], as well as theoretical models of N protein condensation and particle budding [52], demonstrate that the N protein forms multiple distinct clusters around the RNA inside viral particles, with unresolved or empty space between these clusters. Therefore, it is theoretically possible that other host proteins could fit in these spaces and be incorporated into virions based on biophysical properties or due to their location in proximity to sites of budding. Our work lays the foundation for future work by characterizing what may be packaged into viral particles, as well as the specificity of the budding process.

Rapid tools to measure secreted viral particles are needed to address the current and future pandemics. The rapid assay described here provides a broadly applicable reporter to measure coronavirus production that tracks with secreted viral RNA copies and viral infectivity. We further validated the potential of this assay to measure antiviral drugs that reduce viral replication, protein processing, and virion secretion. We posit that this approach will be readily adaptable to detect and screen the inhibitors of future SARS-CoV-2 variants as well as other novel coronaviruses, and will enable the identification of novel targets in coronavirus assembly and release.

## Figures and Tables

**Table 1:** Primer sequences for qRT-PCR.

Primer Name	Sequence
EGFP-qRTPCR-FWD	TCGCCGACCACTACCAGCAGAA
EGFP-qRTPCR-REV	CGCGCTTCTCGTTGGGGTCTTT
NSP15-qRTPCR_FWD	TTTGGGTGTGGACATTGCTGCT
NSP15-qRTPCR_REV	ACAGTGAGTGGTGCACAAATCGT
GAPDH-qRTPCR_FWD	TTCGACAGTCAGCCGCATCTTCTT
GAPDH-qRTPCR_REV	GCCCAATACGACCAAATCCGTTGA
nLuc-12xMBL-FL_FWD	ATGGTCTTCACACTCGAAGATTTCG
nLuc-12xMBL-FL_REV	CAGGTTCAGGGGGAGGTGTG

## Supplementary Materials

The following supporting information can be downloaded at https://www.mdpi.com/article/10.3390/v15061335/s1, Figure S1: Development of SARS-CoV-2 Virus-Like Particles with a nanoLuciferase RNA reporter, Figure S2: Secretion of nanoLuciferase from SARS-CoV-2 infected cells, Figure S3: SARS-CoV-2 and OC43 virions package nano- and renilla- but not firefly luciferase, while VLPs only package nLuc.
