# Supplementary material for: Novel Mode of nanoLuciferase Packaging in SARS-CoV-2 Virions and VLPs Provides Versatile Reporters for Virus Production
Source: Viruses. 2023 Jun 7;15(6):1335. doi: 10.3390/v15061335 (PMC10301038; doi:10.3390/v15061335)
Supplement: Supplementary file 1 [file viruses-15-01335-s001.zip › viruses-2426760-supplementary.pdf]

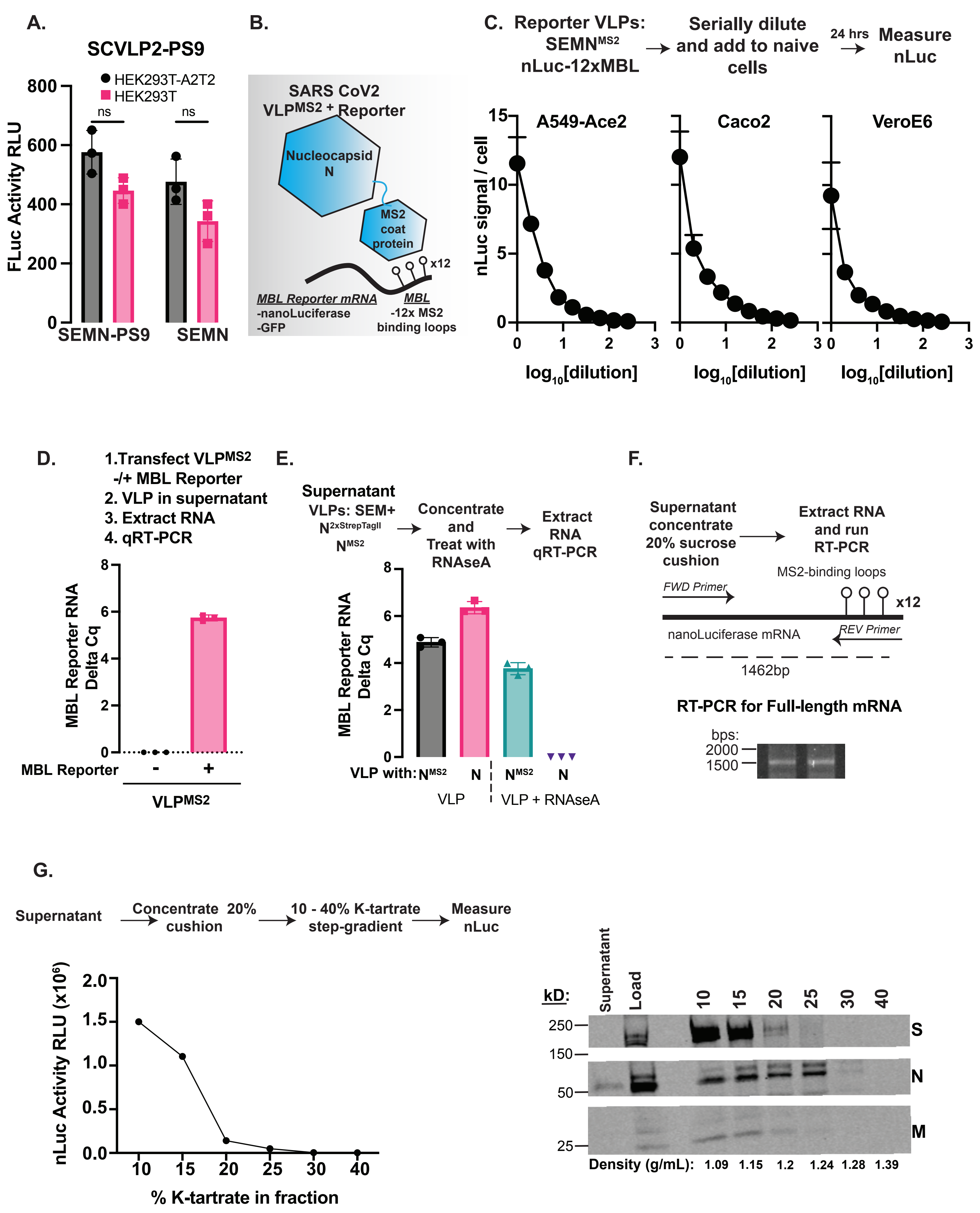

Gullberg et al. Supplemental Figure S1.

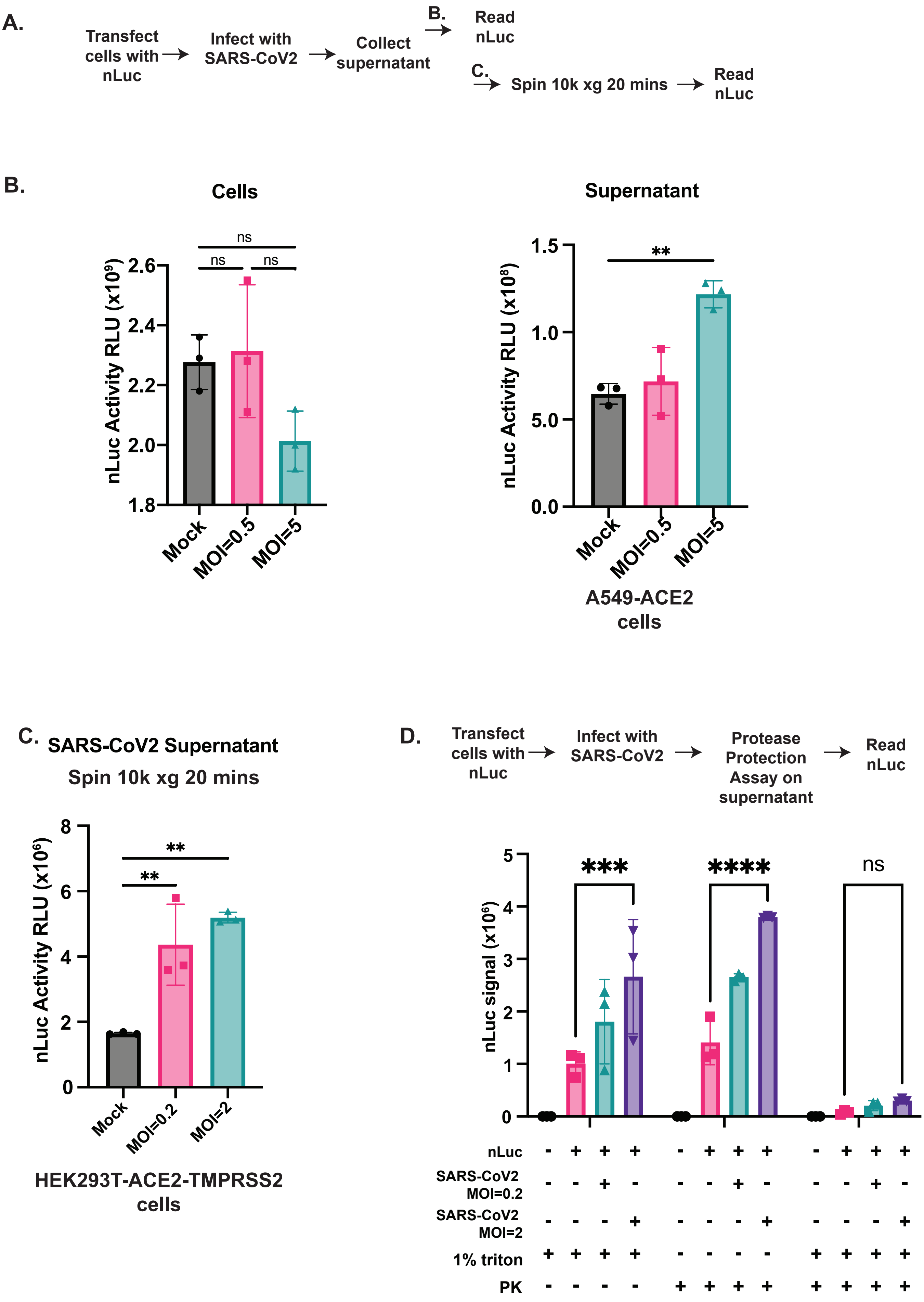

Gullberg et al. Supplemental Figure S2.

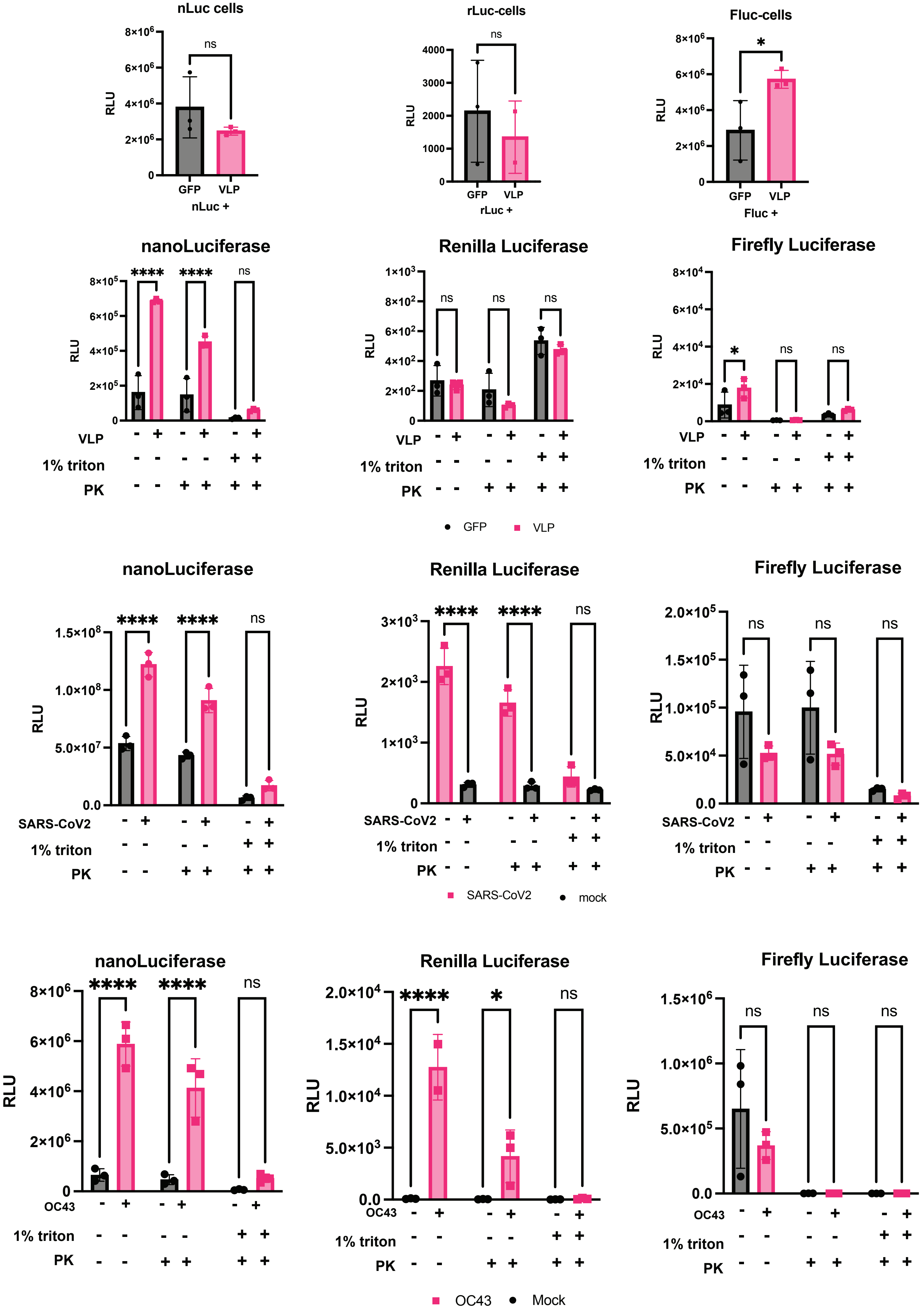

Gullberg et al. Supplemental Figure S3.

Legends to Supplemental Figure

Supplemental Figure S1. Development of SARS-CoV2 Virus Like Particles with a nanoLuciferase RNA reporter

(A) Titration of SCOV2-VLPs with luciferase-PS9 in HEK293T-ACE2/TMPRSS2 and HEK293T cells.

(B) Schematic of trans RNA packaging system. The SARS-CoV2 nucleocapsid (N) protein is genetically fused to the MS2 capsid protein which mediates the specificity of RNA binding. N-MS2 is co-expressed with a plasmid encoding nanoLuciferase (nLuc) and the MS2 binding loops in the 3' region of its RNA. Together with S, E and M, these are expected to form a virus-like particle encapsulating the reporter RNA, which is expressed upon delivery to new cells. The reporter RNA used here: nLuc or GFP mRNA with 12 copies (12x) of the MS2 binding loops.

(C) VLPs can enter naïve cells. VLPs with nLuc-12x were serially diluted and added to naïve indicated cells and incubated for 24 hrs and the nLuc signal was measured.

(D) RNA is secreted with VLPs into the supernatant. VLPs were co-expressed with or without the GFP-12xMBL reporter RNA. qRT-PCR for GFP mRNA show that it is secreted into the supernatant.

(E) The N-MS2 protein is responsible for binding the RNA and RNase A protection. VLPs were produced with the N-MS2 construct or N without a tag and the reporter RNA. VLPs were collected, concentrated and incubated with RNase A to degrade any free RNA. RNase A was inactivated with TRIZOL, RNA was extracted and qRT-PCR was run.

(F) Full-length mRNA is packaged in VLPs. Schematic for RT-PCR of full-length RNA. VLPs with reporter RNA were concentrated and the RNA was extracted and subjected to RT-PCR with primers to amplify the entire construct. The amplified cDNA was run on a 1% agarose gel.

(G) nLuc co-migrates with SARS-CoV2 VLPs in a step-gradient. SARS-CoV2 VLP supernatant was collected from nLuc expressing cells, concentrated through a sucrose cushion and purified on a k-tartrate density step gradient. Each fraction was analyzed for nLuc activity and a western blot for CoV2 structural proteins.

Supplemental Figure S2. Secretion of nanoLuciferase from SARS-CoV2 infected cells.

(A)Schematic of nLuc expression in cells with SARS-CoV2 infection.

(B) A549-ACE2 cells were transfected with nLuc and infected with SARS-CoV2 for 24 hrs and the cells and supernatant were directly read for nLuc signal. *Associated with Figure 2A.*

(C) HET293T-Ace2/TMPRSS2 cells were transfected with nLuc and infected with SARS-CoV2 for 48 hrs and the supernatant was collected, centrifuged at 10kxg for 20 mins to remove large debris and the nLuc activity was measured.

(D) Protease protection assay: nLuc expressing cells were infected with SARS-CoV2 or mock for 48 hrs, after which the supernatant was collected and treated with or without 1% triton followed by PK before measuring the nLuc signal. *Associated with Figure 2A*.

One-way ANOVA with multiple comparisons test was performed: ns=not significant, *=p<0.05, **=p<0.01, ***p<0.001, ****p<0.0001; n=3.

Supplemental Figure S3. SARS-CoV2 and OC43 virions package nano- and renilla- but not firefly luciferase, while VLPs only package nLuc.

Cells expressing nLuc, renilla or firefly luciferases were infected with SARS-CoV2, OC43 co-expressed with SARS-CoV2 VLPs for 48 hrs. The corresponding luciferase signal was measured in the cells. The supernatant was then collected, treated with or without 1% triton, PK, and the luciferase activity measured with the corresponding reagent.

Associated with Figure 3B-D. One-way ANOVA with multiple comparisons test was performed: ns=not significant, *=p<0.05, **=p<0.01, ***p<0.001, ****p<0.0001; n=3.
